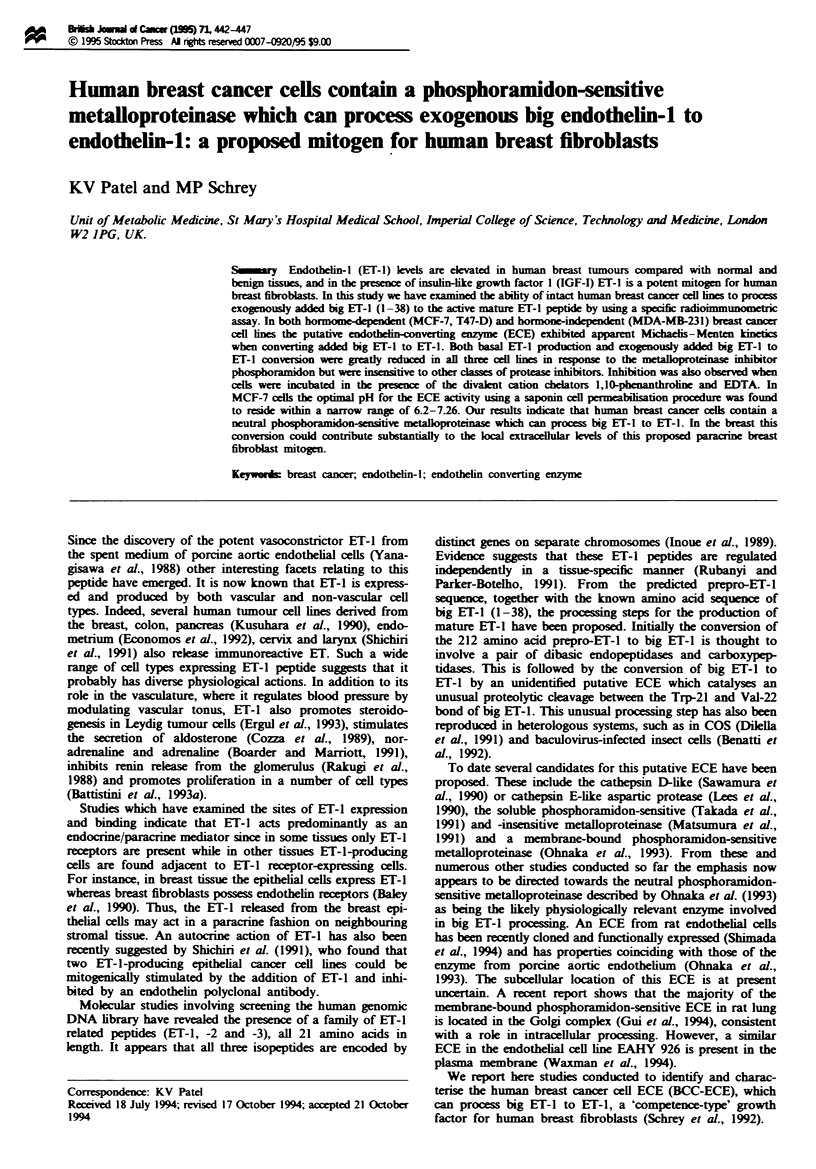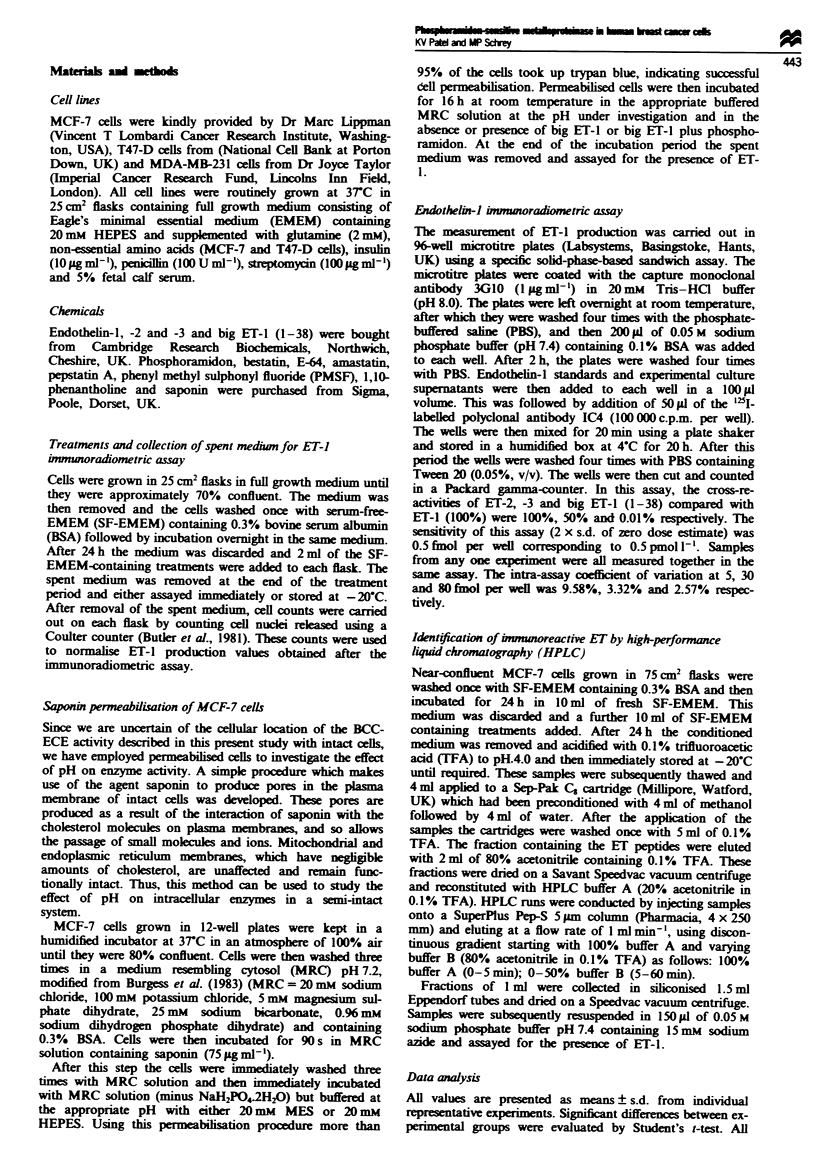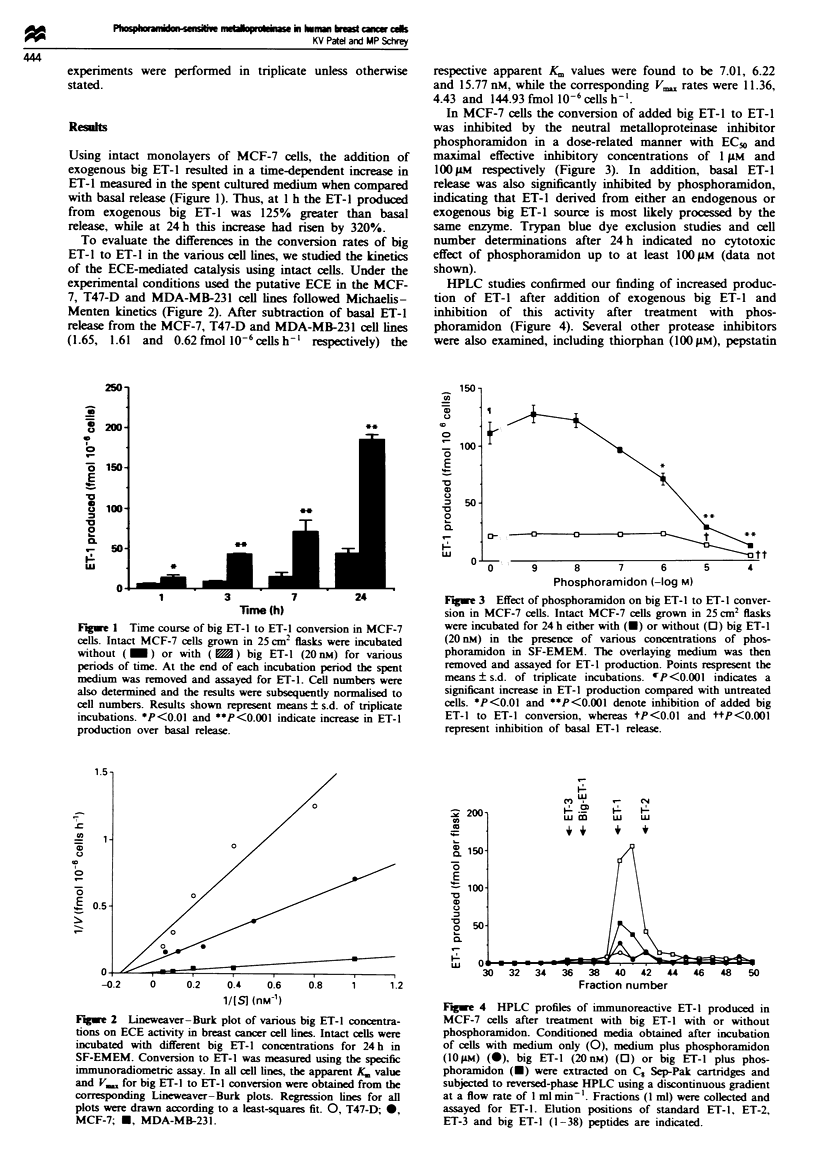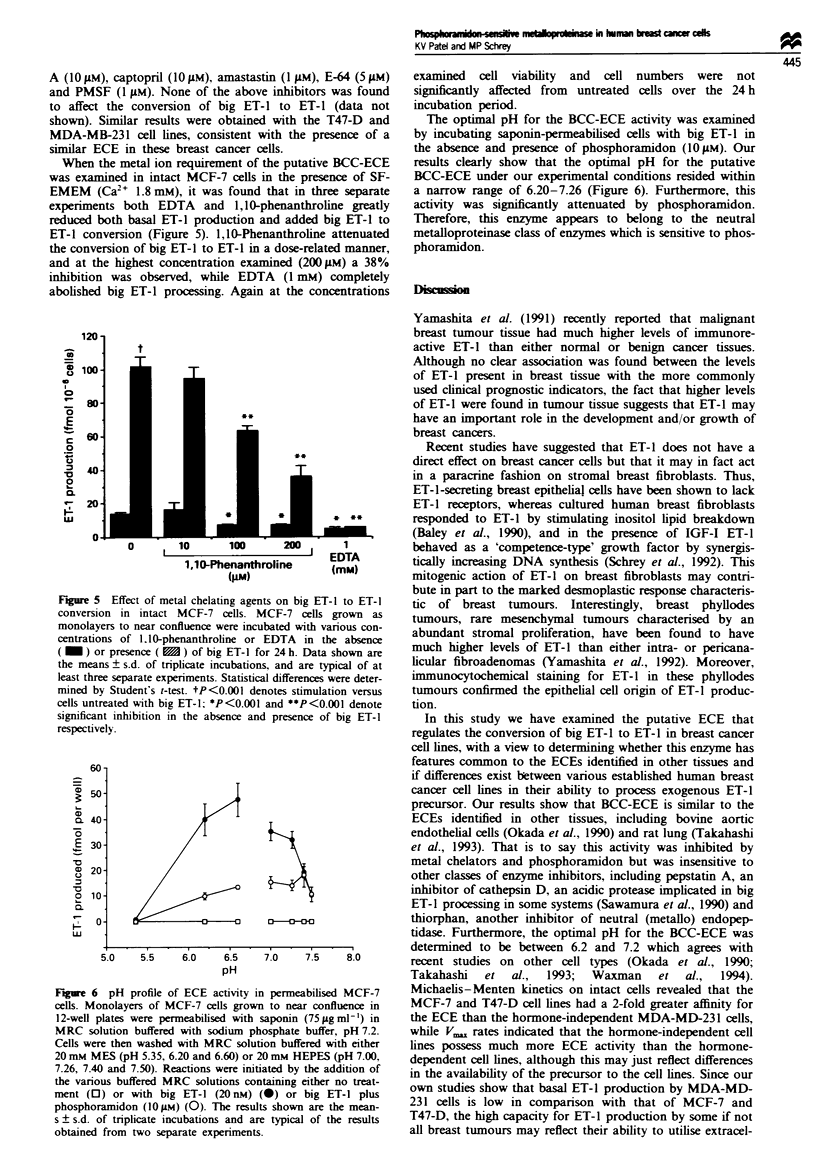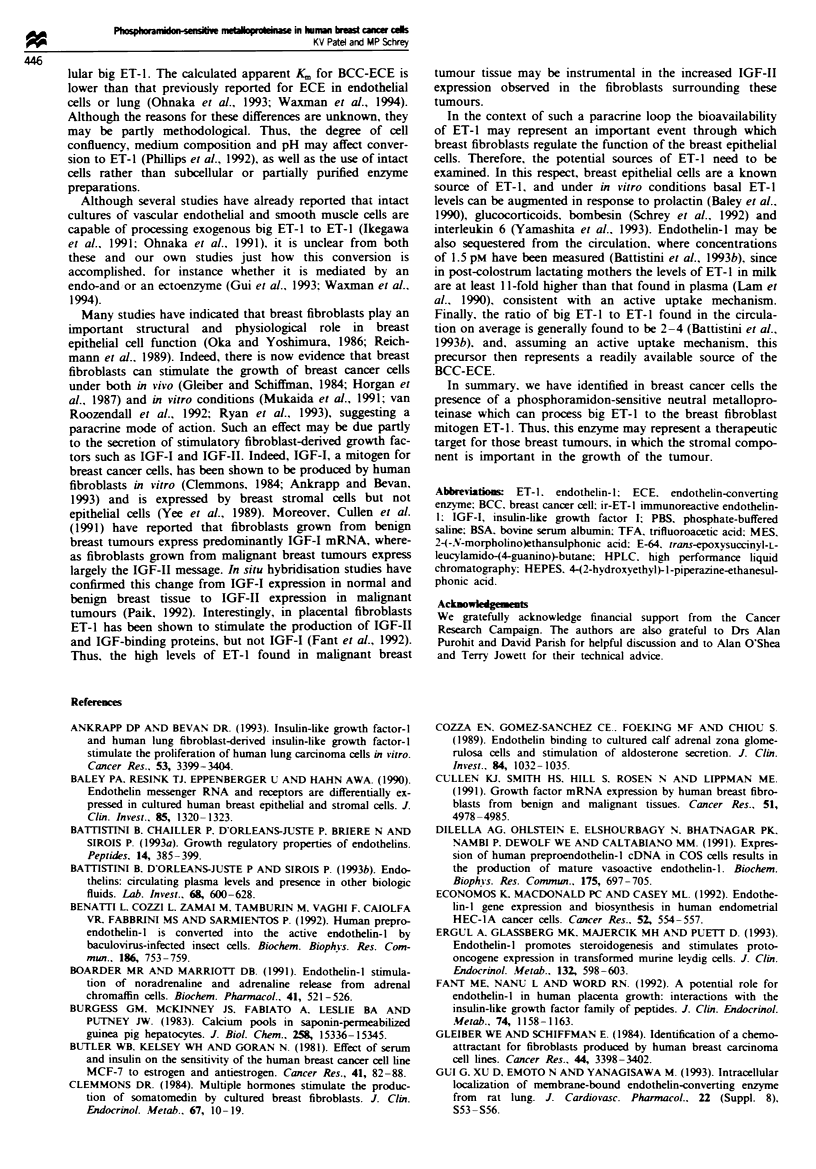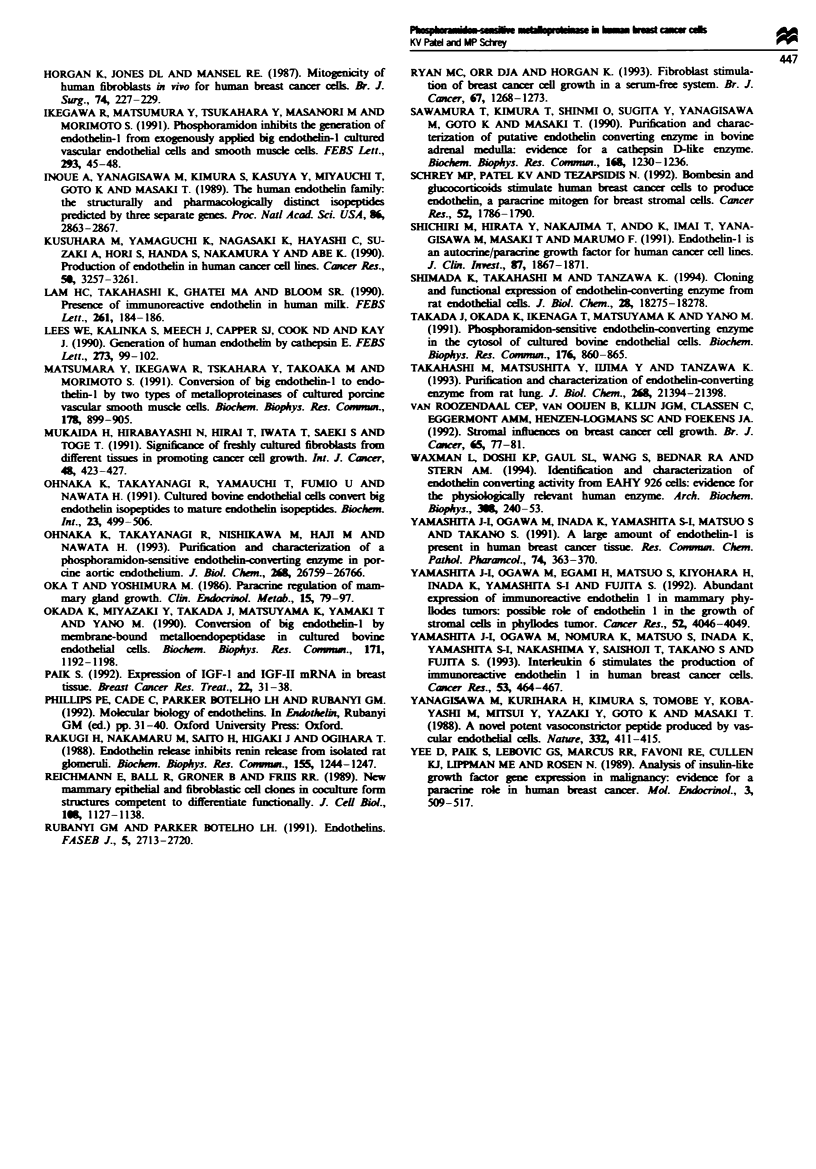# Human breast cancer cells contain a phosphoramidon-sensitive metalloproteinase which can process exogenous big endothelin-1 to endothelin-1: a proposed mitogen for human breast fibroblasts.

**DOI:** 10.1038/bjc.1995.90

**Published:** 1995-03

**Authors:** K. V. Patel, M. P. Schrey

**Affiliations:** Unit of Metabolic Medicine, St Mary's Hospital Medical School, Imperial College of Science, Technology and Medicine, London.

## Abstract

Endothelin-1 (ET-1) levels are elevated in human breast tumours compared with normal and benign tissues, and in the presence of insulin-like growth factor 1 (IGF-I) ET-1 is a potent mitogen for human breast fibroblasts. In this study we have examined the ability of intact human breast cancer cell lines to process exogenously added big ET-1 (1-38) to the active mature ET-1 peptide by using a specific radioimmunometric assay. In both hormome-dependent (MCF-7, T47-D) and hormone-independent (MDA-MB-231) breast cancer cell lines the putative endothelin-converting enzyme (ECE) exhibited apparent Michaelis-Menten kinetics when converting added big ET-1 to ET-1. Both basal ET-1 production and exogenously added big ET-1 to ET-1 conversion were greatly reduced in all three cell lines in response to the metalloproteinase inhibitor phosphoramidon but were insensitive to other classes of protease inhibitors. Inhibition was also observed when cells were incubated in the presence of the divalent cation chelators 1,10-phenanthroline and EDTA. In MCF-7 cells the optimal pH for the ECE activity using a saponin cell permeabilisation procedure was found to residue within a narrow range of 6.2-7.26. Our results indicate that human breast cancer cells contain a neutral phosphoramidon-sensitive metalloproteinase which can process big ET-1 to ET-1. In the breast this conversion could contribute substantially to the local extracellular levels of this proposed paracrine breast fibroblast mitogen.


© 1995 Stockton Press All rights reserved 0007-0920/95 $9.00

### Human breast cancer cells contain a phosphoramidon-sensitive metalloproteinase which can process exogenous big endothelin-1 to endothelin-1: a proposed mitogen for human breast fibroblasts

KV Patel and MP Schrey

*Unit of Metabolic Medicine, St Mary's Hospital Medical School, Imperial College of Science, Technology and Medicine, London W2 1PG, UK.*

**Summary** Endothelin-1 (ET-1) levels are elevated in human breast tumours compared with normal and benign tissues, and in the presence of insulin-like growth factor 1 (IGF-I) ET-1 is a potent mitogen for human breast fibroblasts. In this study we have examined the ability of intact human breast cancer cell lines to process exogenously added big ET-1 (1-38) to the active mature ET-1 peptide by using a specific radioimmunometric assay. In both hormome-dependent (MCF-7, T47-D) and hormone-independent (MDA-MB-231) breast cancer cell lines the putative endothelin-converting enzyme (ECE) exhibited apparent Michaelis-Menten kinetics when converting added big ET-1 to ET-1. Both basal ET-1 production and exogenously added big ET-1 to ET-1 conversion were greatly reduced in all three cell lines in response to the metalloproteinase inhibitor phosphoramidon but were insensitive to other classes of protease inhibitors. Inhibition was also observed when cells were incubated in the presence of the divalent cation chelators 1,10-phenanthroline and EDTA. In MCF-7 cells the optimal pH for the ECE activity using a saponin cell permeabilisation procedure was found to reside within a narrow range of 6.2-7.26. Our results indicate that human breast cancer cells contain a neutral phosphoramidon-sensitive metalloproteinase which can process big ET-1 to ET-1. In the breast this conversion could contribute substantially to the local extracellular levels of this proposed paracrine breast fibroblast mitogen.

**Keywords:** breast cancer; endothelin-1; endothelin converting enzyme

Since the discovery of the potent vasoconstrictor ET-1 from the spent medium of porcine aortic endothelial cells (Yanagisawa *et al.*, 1988) other interesting facets relating to this peptide have emerged. It is now known that ET-1 is expressed and produced by both vascular and non-vascular cell types. Indeed, several human tumour cell lines derived from the breast, colon, pancreas (Kusuhara *et al.*, 1990), endometrium (Economos *et al.*, 1992), cervix and larynx (Shichiri *et al.*, 1991) also release immunoreactive ET. Such a wide range of cell types expressing ET-1 peptide suggests that it probably has diverse physiological actions. In addition to its role in the vasculature, where it regulates blood pressure by modulating vascular tonus, ET-1 also promotes steroidogenesis in Leydig tumour cells (Ergul *et al.*, 1993), stimulates the secretion of aldosterone (Cozza *et al.*, 1989), noradrenaline and adrenaline (Boarder and Marriott, 1991), inhibits renin release from the glomerulus (Rakugi *et al.*, 1988) and promotes proliferation in a number of cell types (Battistini *et al.*, 1993*a*).

Studies which have examined the sites of ET-1 expression and binding indicate that ET-1 acts predominantly as an endocrine/paracrine mediator since in some tissues only ET-1 receptors are present while in other tissues ET-1-producing cells are found adjacent to ET-1 receptor-expressing cells. For instance, in breast tissue the epithelial cells express ET-1 whereas breast fibroblasts possess endothelin receptors (Baley *et al.*, 1990). Thus, the ET-1 released from the breast epithelial cells may act in a paracrine fashion on neighbouring stromal tissue. An autocrine action of ET-1 has also been recently suggested by Shichiri *et al.* (1991), who found that two ET-1-producing epithelial cancer cell lines could be mitogenically stimulated by the addition of ET-1 and inhibited by an endothelin polyclonal antibody.

Molecular studies involving screening the human genomic DNA library have revealed the presence of a family of ET-1 related peptides (ET-1, -2 and -3), all 21 amino acids in length. It appears that all three isopeptides are encoded by distinct genes on separate chromosomes (Inoue *et al.*, 1989). Evidence suggests that these ET-1 peptides are regulated independently in a tissue-specific manner (Rubanyi and Parker-Botelho, 1991). From the predicted prepro-ET-1 sequence, together with the known amino acid sequence of big ET-1 (1-38), the processing steps for the production of mature ET-1 have been proposed. Initially the conversion of the 212 amino acid prepro-ET-1 to big ET-1 is thought to involve a pair of dibasic endopeptidases and carboxypeptidases. This is followed by the conversion of big ET-1 to ET-1 by an unidentified putative ECE which catalyses an unusual proteolytic cleavage between the Trp-21 and Val-22 bond of big ET-1. This unusual processing step has also been reproduced in heterologous systems, such as in COS (Dilella *et al.*, 1991) and baculovirus-infected insect cells (Benatti *et al.*, 1992).

To date several candidates for this putative ECE have been proposed. These include the cathepsin D-like (Sawamura *et al.*, 1990) or cathepsin E-like aspartic protease (Lees *et al.*, 1990), the soluble phosphoramidon-sensitive (Takada *et al.*, 1991) and -insensitive metalloproteinase (Matsumura *et al.*, 1991) and a membrane-bound phosphoramidon-sensitive metalloproteinase (Ohnaka *et al.*, 1993). From these and numerous other studies conducted so far the emphasis now appears to be directed towards the neutral phosphoramidon-sensitive metalloproteinase described by Ohnaka *et al.* (1993) as being the likely physiologically relevant enzyme involved in big ET-1 processing. An ECE from rat endothelial cells has been recently cloned and functionally expressed (Shimada *et al.*, 1994) and has properties coinciding with those of the enzyme from porcine aortic endothelium (Ohnaka *et al.*, 1993). The subcellular location of this ECE is at present uncertain. A recent report shows that the majority of the membrane-bound phosphoramidon-sensitive ECE in rat lung is located in the Golgi complex (Gui *et al.*, 1994), consistent with a role in intracellular processing. However, a similar ECE in the endothelial cell line EAHY 926 is present in the plasma membrane (Waxman *et al.*, 1994).

We report here studies conducted to identify and characterise the human breast cancer cell ECE (BCC-ECE), which can process big ET-1 to ET-1, a 'competence-type' growth factor for human breast fibroblasts (Schrey *et al.*, 1992).

Correspondence: KV Patel
Received 18 July 1994; revised 17 October 1994; accepted 21 October 1994

### Materials and methods

*Cell lines*

MCF-7 cells were kindly provided by Dr Marc Lippman (Vincent T Lombardi Cancer Research Institute, Washington, USA), T47-D cells from (National Cell Bank at Porton Down, UK) and MDA-MB-231 cells from Dr Joyce Taylor (Imperial Cancer Research Fund, Lincolns Inn Field, London). All cell lines were routinely grown at 37°C in 25 $cm^2$ flasks containing full growth medium consisting of Eagle's minimal essential medium (EMEM) containing 20 mM HEPES and supplemented with glutamine (2 mM), non-essential amino acids (MCF-7 and T47-D cells), insulin (10 $\mu g\ ml^{-1}$), penicillin (100 U $ml^{-1}$), streptomycin (100 $\mu g\ ml^{-1}$) and 5% fetal calf serum.

*Chemicals*

Endothelin-1, -2 and -3 and big ET-1 (1–38) were bought from Cambridge Research Biochemicals, Northwich, Cheshire, UK. Phosphoramidon, bestatin, E-64, amastatin, pepstatin A, phenyl methyl sulphonyl fluoride (PMSF), 1,10-phenantholine and saponin were purchased from Sigma, Poole, Dorset, UK.

*Treatments and collection of spent medium for ET-1 immunoradiometric assay*

Cells were grown in 25 $cm^2$ flasks in full growth medium until they were approximately 70% confluent. The medium was then removed and the cells washed once with serum-free-EMEM (SF-EMEM) containing 0.3% bovine serum albumin (BSA) followed by incubation overnight in the same medium. After 24 h the medium was discarded and 2 ml of the SF-EMEM-containing treatments were added to each flask. The spent medium was removed at the end of the treatment period and either assayed immediately or stored at −20°C. After removal of the spent medium, cell counts were carried out on each flask by counting cell nuclei released using a Coulter counter (Butler *et al.*, 1981). These counts were used to normalise ET-1 production values obtained after the immunoradiometric assay.

*Saponin permeabilisation of MCF-7 cells*

Since we are uncertain of the cellular location of the BCC-ECE activity described in this present study with intact cells, we have employed permeabilised cells to investigate the effect of pH on enzyme activity. A simple procedure which makes use of the agent saponin to produce pores in the plasma membrane of intact cells was developed. These pores are produced as a result of the interaction of saponin with the cholesterol molecules on plasma membranes, and so allows the passage of small molecules and ions. Mitochondrial and endoplasmic reticulum membranes, which have negligible amounts of cholesterol, are unaffected and remain functionally intact. Thus, this method can be used to study the effect of pH on intracellular enzymes in a semi-intact system.

MCF-7 cells grown in 12-well plates were kept in a humidified incubator at 37°C in an atmosphere of 100% air until they were 80% confluent. Cells were then washed three times in a medium resembling cytosol (MRC) pH 7.2, modified from Burgess *et al.* (1983) (MRC = 20 mM sodium chloride, 100 mM potassium chloride, 5 mM magnesium sulphate dihydrate, 25 mM sodium bicarbonate, 0.96 mM sodium dihydrogen phosphate dihydrate) and containing 0.3% BSA. Cells were then incubated for 90 s in MRC solution containing saponin (75 $\mu g\ ml^{-1}$).

After this step the cells were immediately washed three times with MRC solution and then immediately incubated with MRC solution (minus $NaH_2PO_4.2H_2O$) but buffered at the appropriate pH with either 20 mM MES or 20 mM HEPES. Using this permeabilisation procedure more than 95% of the cells took up trypan blue, indicating successful cell permeabilisation. Permeabilised cells were then incubated for 16 h at room temperature in the appropriate buffered MRC solution at the pH under investigation and in the absence or presence of big ET-1 or big ET-1 plus phosphoramidon. At the end of the incubation period the spent medium was removed and assayed for the presence of ET-1.

*Endothelin-1 immunoradiometric assay*

The measurement of ET-1 production was carried out in 96-well microtitre plates (Labsystems, Basingstoke, Hants, UK) using a specific solid-phase-based sandwich assay. The microtitre plates were coated with the capture monoclonal antibody 3G10 (1 $\mu g\ ml^{-1}$) in 20 mM Tris–HCl buffer (pH 8.0). The plates were left overnight at room temperature, after which they were washed four times with the phosphate-buffered saline (PBS), and then 200 µl of 0.05 M sodium phosphate buffer (pH 7.4) containing 0.1% BSA was added to each well. After 2 h, the plates were washed four times with PBS. Endothelin-1 standards and experimental culture supernatants were then added to each well in a 100 µl volume. This was followed by addition of 50 µl of the $^{125}I$-labelled polyclonal antibody IC4 (100 000 c.p.m. per well). The wells were then mixed for 20 min using a plate shaker and stored in a humidified box at 4°C for 20 h. After this period the wells were washed four times with PBS containing Tween 20 (0.05%, v/v). The wells were then cut and counted in a Packard gamma-counter. In this assay, the cross-reactivities of ET-2, -3 and big ET-1 (1–38) compared with ET-1 (100%) were 100%, 50% and 0.01% respectively. The sensitivity of this assay (2 × s.d. of zero dose estimate) was 0.5 fmol per well corresponding to 0.5 pmol $l^{-1}$. Samples from any one experiment were all measured together in the same assay. The intra-assay coefficient of variation at 5, 30 and 80 fmol per well was 9.58%, 3.32% and 2.57% respectively.

*Identification of immunoreactive ET by high-performance liquid chromatography (HPLC)*

Near-confluent MCF-7 cells grown in 75 $cm^2$ flasks were washed once with SF-EMEM containing 0.3% BSA and then incubated for 24 h in 10 ml of fresh SF-EMEM. This medium was discarded and a further 10 ml of SF-EMEM containing treatments added. After 24 h the conditioned medium was removed and acidified with 0.1% trifluoroacetic acid (TFA) to pH.4.0 and then immediately stored at −20°C until required. These samples were subsequently thawed and 4 ml applied to a Sep-Pak $C_8$ cartridge (Millipore, Watford, UK) which had been preconditioned with 4 ml of methanol followed by 4 ml of water. After the application of the samples the cartridges were washed once with 5 ml of 0.1% TFA. The fraction containing the ET peptides were eluted with 2 ml of 80% acetonitrile containing 0.1% TFA. These fractions were dried on a Savant Speedvac vacuum centrifuge and reconstituted with HPLC buffer A (20% acetonitrile in 0.1% TFA). HPLC runs were conducted by injecting samples onto a SuperPlus Pep-S 5 µm column (Pharmacia, 4 × 250 mm) and eluting at a flow rate of 1 ml $min^{-1}$, using discontinuous gradient starting with 100% buffer A and varying buffer B (80% acetonitrile in 0.1% TFA) as follows: 100% buffer A (0–5 min); 0–50% buffer B (5–60 min).

Fractions of 1 ml were collected in siliconised 1.5 ml Eppendorf tubes and dried on a Speedvac vacuum centrifuge. Samples were subsequently resuspended in 150 µl of 0.05 M sodium phosphate buffer pH 7.4 containing 15 mM sodium azide and assayed for the presence of ET-1.

*Data analysis*

All values are presented as means ± s.d. from individual representative experiments. Significant differences between experimental groups were evaluated by Student's *t*-test. All experiments were performed in triplicate unless otherwise stated.

### Results

Using intact monolayers of MCF-7 cells, the addition of exogenous big ET-1 resulted in a time-dependent increase in ET-1 measured in the spent cultured medium when compared with basal release (Figure 1). Thus, at 1 h the ET-1 produced from exogenous big ET-1 was 125% greater than basal release, while at 24 h this increase had risen by 320%.

To evaluate the differences in the conversion rates of big ET-1 to ET-1 in the various cell lines, we studied the kinetics of the ECE-mediated catalysis using intact cells. Under the experimental conditions used the putative ECE in the MCF-7, T47-D and MDA-MB-231 cell lines followed Michaelis–Menten kinetics (Figure 2). After subtraction of basal ET-1 release from the MCF-7, T47-D and MDA-MB-231 cell lines (1.65, 1.61 and 0.62 fmol $10^{-6}$ cells $h^{-1}$ respectively) the respective apparent $K_m$ values were found to be 7.01, 6.22 and 15.77 nM, while the corresponding $V_{max}$ rates were 11.36, 4.43 and 144.93 fmol $10^{-6}$ cells $h^{-1}$.

In MCF-7 cells the conversion of added big ET-1 to ET-1 was inhibited by the neutral metalloproteinase inhibitor phosphoramidon in a dose-related manner with $EC_{50}$ and maximal effective inhibitory concentrations of 1 μM and 100 μM respectively (Figure 3). In addition, basal ET-1 release was also significantly inhibited by phosphoramidon, indicating that ET-1 derived from either an endogenous or exogenous big ET-1 source is most likely processed by the same enzyme. Trypan blue dye exclusion studies and cell number determinations after 24 h indicated no cytotoxic effect of phosphoramidon up to at least 100 μM (data not shown).

HPLC studies confirmed our finding of increased production of ET-1 after addition of exogenous big ET-1 and inhibition of this activity after treatment with phosphoramidon (Figure 4). Several other protease inhibitors were also examined, including thiorphan (100 μM), pepstatin

**Figure 1** Time course of big ET-1 to ET-1 conversion in MCF-7 cells. Intact MCF-7 cells grown in 25 cm$^2$ flasks were incubated without (■) or with (▨) big ET-1 (20 nM) for various periods of time. At the end of each incubation period the spent medium was removed and assayed for ET-1. Cell numbers were also determined and the results were subsequently normalised to cell numbers. Results shown represent means ± s.d. of triplicate incubations. *$P<0.01$ and **$P<0.001$ indicate increase in ET-1 production over basal release.

**Figure 3** Effect of phosphoramidon on big ET-1 to ET-1 conversion in MCF-7 cells. Intact MCF-7 cells grown in 25 cm$^2$ flasks were incubated for 24 h either with (■) or without (□) big ET-1 (20 nM) in the presence of various concentrations of phosphoramidon in SF-EMEM. The overlaying medium was then removed and assayed for ET-1 production. Points respresent the means ± s.d. of triplicate incubations. ¶$P<0.001$ indicates a significant increase in ET-1 production compared with untreated cells. *$P<0.01$ and **$P<0.001$ denote inhibition of added big ET-1 to ET-1 conversion, whereas †$P<0.01$ and ††$P<0.001$ represent inhibition of basal ET-1 release.

**Figure 2** Lineweaver–Burk plot of various big ET-1 concentrations on ECE activity in breast cancer cell lines. Intact cells were incubated with different big ET-1 concentrations for 24 h in SF-EMEM. Conversion to ET-1 was measured using the specific immunoradiometric assay. In all cell lines, the apparent $K_m$ value and $V_{max}$ for big ET-1 to ET-1 conversion were obtained from the corresponding Lineweaver–Burk plots. Regression lines for all plots were drawn according to a least-squares fit. ○, T47-D; ●, MCF-7; ■, MDA-MB-231.

**Figure 4** HPLC profiles of immunoreactive ET-1 produced in MCF-7 cells after treatment with big ET-1 with or without phosphoramidon. Conditioned media obtained after incubation of cells with medium only (○), medium plus phosphoramidon (10 μM) (●), big ET-1 (20 nM) (□) or big ET-1 plus phosphoramidon (■) were extracted on $C_8$ Sep-Pak cartridges and subjected to reversed-phase HPLC using a discontinuous gradient at a flow rate of 1 ml min$^{-1}$. Fractions (1 ml) were collected and assayed for ET-1. Elution positions of standard ET-1, ET-2, ET-3 and big ET-1 (1–38) peptides are indicated.

A (10 μM), captopril (10 μM), amastastin (1 μM), E-64 (5 μM) and PMSF (1 μM). None of the above inhibitors was found to affect the conversion of big ET-1 to ET-1 (data not shown). Similar results were obtained with the T47-D and MDA-MB-231 cell lines, consistent with the presence of a similar ECE in these breast cancer cells.

When the metal ion requirement of the putative BCC-ECE was examined in intact MCF-7 cells in the presence of SF-EMEM ($Ca^{2+}$ 1.8 mM), it was found that in three separate experiments both EDTA and 1,10-phenanthroline greatly reduced both basal ET-1 production and added big ET-1 to ET-1 conversion (Figure 5). 1,10-Phenanthroline attenuated the conversion of big ET-1 to ET-1 in a dose-related manner, and at the highest concentration examined (200 μM) a 38% inhibition was observed, while EDTA (1 mM) completely abolished big ET-1 processing. Again at the concentrations

**Figure 5** Effect of metal chelating agents on big ET-1 to ET-1 conversion in intact MCF-7 cells. MCF-7 cells grown as monolayers to near confluence were incubated with various concentrations of 1,10-phenanthroline or EDTA in the absence (■) or presence (▨) of big ET-1 for 24 h. Data shown are the means ± s.d. of triplicate incubations, and are typical of at least three separate experiments. Statistical differences were determined by Student's *t*-test. †$P<0.001$ denotes stimulation versus cells untreated with big ET-1; *$P<0.001$ and **$P<0.001$ denote significant inhibition in the absence and presence of big ET-1 respectively.

**Figure 6** pH profile of ECE activity in permeabilised MCF-7 cells. Monolayers of MCF-7 cells grown to near confluence in 12-well plates were permeabilised with saponin (75 μg $ml^{-1}$) in MRC solution buffered with sodium phosphate buffer, pH 7.2. Cells were then washed with MRC solution buffered with either 20 mM MES (pH 5.35, 6.20 and 6.60) or 20 mM HEPES (pH 7.00, 7.26, 7.40 and 7.50). Reactions were initiated by the addition of the various buffered MRC solutions containing either no treatment (□) or with big ET-1 (20 nM) (●) or big ET-1 plus phosphoramidon (10 μM) (○). The results shown are the means ± s.d. of triplicate incubations and are typical of the results obtained from two separate experiments.

examined cell viability and cell numbers were not significantly affected from untreated cells over the 24 h incubation period.

The optimal pH for the BCC-ECE activity was examined by incubating saponin-permeabilised cells with big ET-1 in the absence and presence of phosphoramidon (10 μM). Our results clearly show that the optimal pH for the putative BCC-ECE under our experimental conditions resided within a narrow range of 6.20–7.26 (Figure 6). Furthermore, this activity was significantly attenuated by phosphoramidon. Therefore, this enzyme appears to belong to the neutral metalloproteinase class of enzymes which is sensitive to phosphoramidon.

### Discussion

Yamashita *et al.* (1991) recently reported that malignant breast tumour tissue had much higher levels of immunoreactive ET-1 than either normal or benign cancer tissues. Although no clear association was found between the levels of ET-1 present in breast tissue with the more commonly used clinical prognostic indicators, the fact that higher levels of ET-1 were found in tumour tissue suggests that ET-1 may have an important role in the development and/or growth of breast cancers.

Recent studies have suggested that ET-1 does not have a direct effect on breast cancer cells but that it may in fact act in a paracrine fashion on stromal breast fibroblasts. Thus, ET-1-secreting breast epithelial cells have been shown to lack ET-1 receptors, whereas cultured human breast fibroblasts responded to ET-1 by stimulating inositol lipid breakdown (Baley *et al.*, 1990), and in the presence of IGF-I ET-1 behaved as a 'competence-type' growth factor by synergistically increasing DNA synthesis (Schrey *et al.*, 1992). This mitogenic action of ET-1 on breast fibroblasts may contribute in part to the marked desmoplastic response characteristic of breast tumours. Interestingly, breast phyllodes tumours, rare mesenchymal tumours characterised by an abundant stromal proliferation, have been found to have much higher levels of ET-1 than either intra- or pericanalicular fibroadenomas (Yamashita *et al.*, 1992). Moreover, immunocytochemical staining for ET-1 in these phyllodes tumours confirmed the epithelial cell origin of ET-1 production.

In this study we have examined the putative ECE that regulates the conversion of big ET-1 to ET-1 in breast cancer cell lines, with a view to determining whether this enzyme has features common to the ECEs identified in other tissues and if differences exist between various established human breast cancer cell lines in their ability to process exogenous ET-1 precursor. Our results show that BCC-ECE is similar to the ECEs identified in other tissues, including bovine aortic endothelial cells (Okada *et al.*, 1990) and rat lung (Takahashi *et al.*, 1993). That is to say this activity was inhibited by metal chelators and phosphoramidon but was insensitive to other classes of enzyme inhibitors, including pepstatin A, an inhibitor of cathepsin D, an acidic protease implicated in big ET-1 processing in some systems (Sawamura *et al.*, 1990) and thiorphan, another inhibitor of neutral (metallo) endopeptidase. Furthermore, the optimal pH for the BCC-ECE was determined to be between 6.2 and 7.2 which agrees with recent studies on other cell types (Okada *et al.*, 1990; Takahashi *et al.*, 1993; Waxman *et al.*, 1994). Michaelis–Menten kinetics on intact cells revealed that the MCF-7 and T47-D cell lines had a 2-fold greater affinity for the ECE than the hormone-independent MDA-MD-231 cells, while $V_{max}$ rates indicated that the hormone-independent cell lines possess much more ECE activity than the hormone-dependent cell lines, although this may just reflect differences in the availability of the precursor to the cell lines. Since our own studies show that basal ET-1 production by MDA-MD-231 cells is low in comparison with that of MCF-7 and T47-D, the high capacity for ET-1 production by some if not all breast tumours may reflect their ability to utilise extracellular big ET-1. The calculated apparent $K_m$ for BCC-ECE is lower than that previously reported for ECE in endothelial cells or lung (Ohnaka *et al.*, 1993; Waxman *et al.*, 1994). Although the reasons for these differences are unknown, they may be partly methodological. Thus, the degree of cell confluency, medium composition and pH may affect conversion to ET-1 (Phillips *et al.*, 1992), as well as the use of intact cells rather than subcellular or partially purified enzyme preparations.

Although several studies have already reported that intact cultures of vascular endothelial and smooth muscle cells are capable of processing exogenous big ET-1 to ET-1 (Ikegawa *et al.*, 1991; Ohnaka *et al.*, 1991), it is unclear from both these and our own studies just how this conversion is accomplished, for instance whether it is mediated by an endo-and/or an ectoenzyme (Gui *et al.*, 1993; Waxman *et al.*, 1994).

Many studies have indicated that breast fibroblasts play an important structural and physiological role in breast epithelial cell function (Oka and Yoshimura, 1986; Reichmann *et al.*, 1989). Indeed, there is now evidence that breast fibroblasts can stimulate the growth of breast cancer cells under both *in vivo* (Gleiber and Schiffman, 1984; Horgan *et al.*, 1987) and *in vitro* conditions (Mukaida *et al.*, 1991; van Roozendall *et al.*, 1992; Ryan *et al.*, 1993), suggesting a paracrine mode of action. Such an effect may be due partly to the secretion of stimulatory fibroblast-derived growth factors such as IGF-I and IGF-II. Indeed, IGF-I, a mitogen for breast cancer cells, has been shown to be produced by human fibroblasts *in vitro* (Clemmons, 1984; Ankrapp and Bevan, 1993) and is expressed by breast stromal cells but not epithelial cells (Yee *et al.*, 1989). Moreover, Cullen *et al.* (1991) have reported that fibroblasts grown from benign breast tumours express predominantly IGF-I mRNA, whereas fibroblasts grown from malignant breast tumours express largely the IGF-II message. *In situ* hybridisation studies have confirmed this change from IGF-I expression in normal and benign breast tissue to IGF-II expression in malignant tumours (Paik, 1992). Interestingly, in placental fibroblasts ET-1 has been shown to stimulate the production of IGF-II and IGF-binding proteins, but not IGF-I (Fant *et al.*, 1992). Thus, the high levels of ET-1 found in malignant breast tumour tissue may be instrumental in the increased IGF-II expression observed in the fibroblasts surrounding these tumours.

In the context of such a paracrine loop the bioavailability of ET-1 may represent an important event through which breast fibroblasts regulate the function of the breast epithelial cells. Therefore, the potential sources of ET-1 need to be examined. In this respect, breast epithelial cells are a known source of ET-1, and under *in vitro* conditions basal ET-1 levels can be augmented in response to prolactin (Baley *et al.*, 1990), glucocorticoids, bombesin (Schrey *et al.*, 1992) and interleukin 6 (Yamashita *et al.*, 1993). Endothelin-1 may be also sequestered from the circulation, where concentrations of 1.5 pM have been measured (Battistini *et al.*, 1993*b*), since in post-colostrum lactating mothers the levels of ET-1 in milk are at least 11-fold higher than that found in plasma (Lam *et al.*, 1990), consistent with an active uptake mechanism. Finally, the ratio of big ET-1 to ET-1 found in the circulation on average is generally found to be 2–4 (Battistini *et al.*, 1993*b*), and, assuming an active uptake mechanism, this precursor then represents a readily available source of the BCC-ECE.

In summary, we have identified in breast cancer cells the presence of a phosphoramidon-sensitive neutral metalloproteinase which can process big ET-1 to the breast fibroblast mitogen ET-1. Thus, this enzyme may represent a therapeutic target for those breast tumours, in which the stromal component is important in the growth of the tumour.

**Abbreviations:** ET-1, endothelin-1; ECE, endothelin-converting enzyme; BCC, breast cancer cell; ir-ET-1 immunoreactive endothelin-1; IGF-I, insulin-like growth factor I; PBS, phosphate-buffered saline; BSA, bovine serum albumin; TFA, trifluoroacetic acid; MES, 2-(-*N*-morpholino)ethansulphonic acid; E-64, *trans*-epoxysuccinyl-L-leucylamido-(4-guanino)-butane; HPLC, high performance liquid chromatography; HEPES, 4-(2-hydroxyethyl)-1-piperazine-ethanesulphonic acid.

**Acknowledgements**
We gratefully acknowledge financial support from the Cancer Research Campaign. The authors are also grateful to Drs Alan Purohit and David Parish for helpful discussion and to Alan O'Shea and Terry Jowett for their technical advice.

### References

ANKRAPP DP AND BEVAN DR. (1993). Insulin-like growth factor-1 and human lung fibroblast-derived insulin-like growth factor-1 stimulate the proliferation of human lung carcinoma cells *in vitro*. *Cancer Res.*, **53**, 3399–3404.

BALEY PA, RESINK TJ, EPPENBERGER U AND HAHN AWA. (1990). Endothelin messenger RNA and receptors are differentially expressed in cultured human breast epithelial and stromal cells. *J. Clin. Invest.*, **85**, 1320–1323.

BATTISTINI B, CHAILLER P, D'ORLEANS-JUSTE P, BRIERE N AND SIROIS P. (1993*a*). Growth regulatory properties of endothelins. *Peptides*, **14**, 385–399.

BATTISTINI B, D'ORLEANS-JUSTE P AND SIROIS P. (1993*b*). Endothelins: circulating plasma levels and presence in other biologic fluids. *Lab. Invest.*, **68**, 600–628.

BENATTI L, COZZI L, ZAMAI M, TAMBURIN M, VAGHI F, CAIOLFA VR, FABBRINI MS AND SARMIENTOS P. (1992). Human preproendothelin-1 is converted into the active endothelin-1 by baculovirus-infected insect cells. *Biochem. Biophys. Res. Commun.*, **186**, 753–759.

BOARDER MR AND MARRIOTT DB. (1991). Endothelin-1 stimulation of noradrenaline and adrenaline release from adrenal chromaffin cells. *Biochem. Pharmacol.*, **41**, 521–526.

BURGESS GM, MCKINNEY JS, FABIATO A, LESLIE BA AND PUTNEY JW. (1983). Calcium pools in saponin-permeabilized guinea pig hepatocytes. *J. Biol. Chem.*, **258**, 15336–15345.

BUTLER WB, KELSEY WH AND GORAN N. (1981). Effect of serum and insulin on the sensitivity of the human breast cancer cell line MCF-7 to estrogen and antiestrogen. *Cancer Res.*, **41**, 82–88.

CLEMMONS DR. (1984). Multiple hormones stimulate the production of somatomedin by cultured breast fibroblasts. *J. Clin. Endocrinol. Metab.*, **67**, 10–19.

COZZA EN, GOMEZ-SANCHEZ CE., FOEKING MF AND CHIOU S. (1989). Endothelin binding to cultured calf adrenal zona glomerulosa cells and stimulation of aldosterone secretion. *J. Clin. Invest.*, **84**, 1032–1035.

CULLEN KJ, SMITH HS, HILL S, ROSEN N AND LIPPMAN ME. (1991). Growth factor mRNA expression by human breast fibroblasts from benign and malignant tissues. *Cancer Res.*, **51**, 4978–4985.

DILELLA AG, OHLSTEIN E, ELSHOURBAGY N, BHATNAGAR PK, NAMBI P, DEWOLF WE AND CALTABIANO MM. (1991). Expression of human preproendothelin-1 cDNA in COS cells results in the production of mature vasoactive endothelin-1. *Biochem. Biophys. Res. Commun.*, **175**, 697–705.

ECONOMOS K, MACDONALD PC AND CASEY ML. (1992). Endothelin-1 gene expression and biosynthesis in human endometrial HEC-1A cancer cells. *Cancer Res.*, **52**, 554–557.

ERGUL A, GLASSBERG MK, MAJERCIK MH AND PUETT D. (1993). Endothelin-1 promotes steroidogenesis and stimulates protooncogene expression in transformed murine leydig cells. *J. Clin. Endocrinol. Metab.*, **132**, 598–603.

FANT ME, NANU L AND WORD RN. (1992). A potential role for endothelin-1 in human placenta growth: interactions with the insulin-like growth factor family of peptides. *J. Clin. Endocrinol. Metab.*, **74**, 1158–1163.

GLEIBER WE AND SCHIFFMAN E. (1984). Identification of a chemoattractant for fibroblasts produced by human breast carcinoma cell lines. *Cancer Res.*, **44**, 3398–3402.

GUI G, XU D, EMOTO N AND YANAGISAWA M. (1993). Intracellular localization of membrane-bound endothelin-converting enzyme from rat lung. *J. Cardiovasc. Pharmacol.*, **22** (Suppl. 8), S53–S56.

HORGAN K, JONES DL AND MANSEL RE. (1987). Mitogenicity of human fibroblasts *in vivo* for human breast cancer cells. *Br. J. Surg.*, **74**, 227–229.

IKEGAWA R, MATSUMURA Y, TSUKAHARA Y, MASANORI M AND MORIMOTO S. (1991). Phosphoramidon inhibits the generation of endothelin-1 from exogenously applied big endothelin-1 cultured vascular endothelial cells and smooth muscle cells. *FEBS Lett.*, **293**, 45–48.

INOUE A, YANAGISAWA M, KIMURA S, KASUYA Y, MIYAUCHI T, GOTO K AND MASAKI T. (1989). The human endothelin family: the structurally and pharmacologically distinct isopeptides predicted by three separate genes. *Proc. Natl Acad. Sci. USA*, **86**, 2863–2867.

KUSUHARA M, YAMAGUCHI K, NAGASAKI K, HAYASHI C, SUZAKI A, HORI S, HANDA S, NAKAMURA Y AND ABE K. (1990). Production of endothelin in human cancer cell lines. *Cancer Res.*, **50**, 3257–3261.

LAM HC, TAKAHASHI K, GHATEI MA AND BLOOM SR. (1990). Presence of immunoreactive endothelin in human milk. *FEBS Lett.*, **261**, 184–186.

LEES WE, KALINKA S, MEECH J, CAPPER SJ, COOK ND AND KAY J. (1990). Generation of human endothelin by cathepsin E. *FEBS Lett.*, **273**, 99–102.

MATSUMARA Y, IKEGAWA R, TSKAHARA Y, TAKOAKA M AND MORIMOTO S. (1991). Conversion of big endothelin-1 to endothelin-1 by two types of metalloproteinases of cultured porcine vascular smooth muscle cells. *Biochem. Biophys. Res. Commun.*, **178**, 899–905.

MUKAIDA H, HIRABAYASHI N, HIRAI T, IWATA T, SAEKI S AND TOGE T. (1991). Significance of freshly cultured fibroblasts from different tissues in promoting cancer cell growth. *Int. J. Cancer*, **48**, 423–427.

OHNAKA K, TAKAYANAGI R, YAMAUCHI T, FUMIO U AND NAWATA H. (1991). Cultured bovine endothelial cells convert big endothelin isopeptides to mature endothelin isopeptides. *Biochem. Int.*, **23**, 499–506.

OHNAKA K, TAKAYANAGI R, NISHIKAWA M, HAJI M AND NAWATA H. (1993). Purification and characterization of a phosphoramidon-sensitive endothelin-converting enzyme in porcine aortic endothelium. *J. Biol. Chem.*, **268**, 26759–26766.

OKA T AND YOSHIMURA M. (1986). Paracrine regulation of mammary gland growth. *Clin. Endocrinol. Metab.*, **15**, 79–97.

OKADA K, MIYAZAKI Y, TAKADA J, MATSUYAMA K, YAMAKI T AND YANO M. (1990). Conversion of big endothelin-1 by membrane-bound metalloendopeptidase in cultured bovine endothelial cells. *Biochem. Biophys. Res. Commun.*, **171**, 1192–1198.

PAIK S. (1992). Expression of IGF-I and IGF-II mRNA in breast tissue. *Breast Cancer Res. Treat.*, **22**, 31–38.

PHILLIPS PE, CADE C, PARKER BOTELHO LH AND RUBANYI GM. (1992). Molecular biology of endothelins. In *Endothelin*, Rubanyi GM (ed.) pp. 31–40. Oxford University Press: Oxford.

RAKUGI H, NAKAMARU M, SAITO H, HIGAKI J AND OGIHARA T. (1988). Endothelin release inhibits renin release from isolated rat glomeruli. *Biochem. Biophys. Res. Commun.*, **155**, 1244–1247.

REICHMANN E, BALL R, GRONER B AND FRIIS RR. (1989). New mammary epithelial and fibroblastic cell clones in coculture form structures competent to differentiate functionally. *J. Cell Biol.*, **108**, 1127–1138.

RUBANYI GM AND PARKER BOTELHO LH. (1991). Endothelins. *FASEB J.*, **5**, 2713–2720.

RYAN MC, ORR DJA AND HORGAN K. (1993). Fibroblast stimulation of breast cancer cell growth in a serum-free system. *Br. J. Cancer*, **67**, 1268–1273.

SAWAMURA T, KIMURA T, SHINMI O, SUGITA Y, YANAGISAWA M, GOTO K AND MASAKI T. (1990). Purification and characterization of putative endothelin converting enzyme in bovine adrenal medulla: evidence for a cathepsin D-like enzyme. *Biochem. Biophys. Res. Commun.*, **168**, 1230–1236.

SCHREY MP, PATEL KV AND TEZAPSIDIS N. (1992). Bombesin and glucocorticoids stimulate human breast cancer cells to produce endothelin, a paracrine mitogen for breast stromal cells. *Cancer Res.*, **52**, 1786–1790.

SHICHIRI M, HIRATA Y, NAKAJIMA T, ANDO K, IMAI T, YANAGISAWA M, MASAKI T AND MARUMO F. (1991). Endothelin-1 is an autocrine/paracrine growth factor for human cancer cell lines. *J. Clin. Invest.*, **87**, 1867–1871.

SHIMADA K, TAKAHASHI M AND TANZAWA K. (1994). Cloning and functional expression of endothelin-converting enzyme from rat endothelial cells. *J. Biol. Chem.*, **28**, 18275–18278.

TAKADA J, OKADA K, IKENAGA T, MATSUYAMA K AND YANO M. (1991). Phosphoramidon-sensitive endothelin-converting enzyme in the cytosol of cultured bovine endothelial cells. *Biochem. Biophys. Res. Commun.*, **176**, 860–865.

TAKAHASHI M, MATSUSHITA Y, IIJIMA Y AND TANZAWA K. (1993). Purification and characterization of endothelin-converting enzyme from rat lung. *J. Biol. Chem.*, **268**, 21394–21398.

VAN ROOZENDAAL CEP, VAN OOIJEN B, KLIJN JGM, CLASSEN C, EGGERMONT AMM, HENZEN-LOGMANS SC AND FOEKENS JA. (1992). Stromal influences on breast cancer cell growth. *Br. J. Cancer*, **65**, 77–81.

WAXMAN L, DOSHI KP, GAUL SL, WANG S, BEDNAR RA AND STERN AM. (1994). Identification and characterization of endothelin converting activity from EAHY 926 cells: evidence for the physiologically relevant human enzyme. *Arch. Biochem. Biophys.*, **308**, 240–53.

YAMASHITA J-I, OGAWA M, INADA K, YAMASHITA S-I, MATSUO S AND TAKANO S. (1991). A large amount of endothelin-1 is present in human breast cancer tissue. *Res. Commun. Chem. Pathol. Pharamcol.*, **74**, 363–370.

YAMASHITA J-I, OGAWA M, EGAMI H, MATSUO S, KIYOHARA H, INADA K, YAMASHITA S-I AND FUJITA S. (1992). Abundant expression of immunoreactive endothelin 1 in mammary phyllodes tumors: possible role of endothelin 1 in the growth of stromal cells in phyllodes tumor. *Cancer Res.*, **52**, 4046–4049.

YAMASHITA J-I, OGAWA M, NOMURA K, MATSUO S, INADA K, YAMASHITA S-I, NAKASHIMA Y, SAISHOJI T, TAKANO S AND FUJITA S. (1993). Interleukin 6 stimulates the production of immunoreactive endothelin 1 in human breast cancer cells. *Cancer Res.*, **53**, 464–467.

YANAGISAWA M, KURIHARA H, KIMURA S, TOMOBE Y, KOBAYASHI M, MITSUI Y, YAZAKI Y, GOTO K AND MASAKI T. (1988). A novel potent vasoconstrictor peptide produced by vascular endothelial cells. *Nature*, **332**, 411–415.

YEE D, PAIK S, LEBOVIC GS, MARCUS RR, FAVONI RE, CULLEN KJ, LIPPMAN ME AND ROSEN N. (1989). Analysis of insulin-like growth factor gene expression in malignancy: evidence for a paracrine role in human breast cancer. *Mol. Endocrinol.*, **3**, 509–517.